# Chemical Constituents, Antioxidant, Anti-Tyrosinase, Cytotoxicity, and Anti-Melanogenesis Activities of *Etlingera elatior* (Jack) Leaf Essential Oils

**DOI:** 10.3390/molecules27113469

**Published:** 2022-05-27

**Authors:** Sarita Sangthong, Itthayakorn Promputtha, Punyawatt Pintathong, Phanuphong Chaiwut

**Affiliations:** 1 School of Cosmetic Science, Mae Fah Luang University, Chiang Rai 57100, Thailand; punyawatt.pin@mfu.ac.th (P.P.); phanuphong@mfu.ac.th (P.C.); 2 Green Cosmetic Technology Research Group, Mae Fah Luang University, Chiang Rai 57100, Thailand; 3 Department of Biology, Faculty of Science, Chiang Mai University, Chiang Mai 50200, Thailand; itthayakorn.p@cmu.ac.th; 4 Research Center in Bioresources for Agriculture, Industry and Medicine, Chiang Mai University, Chiang Mai 50200, Thailand

**Keywords:** torch ginger, terpenes, antioxidant, hydrodistillation, microwave assisted, melanin content

## Abstract

Essential oils of plants have been used widely in cosmetic preparations. Being both perfuming and active ingredients, the functions of essential oils mean they are high-value ingredients. In this study, the leaf of *Etlingera elatior* (Jack) or Torch ginger was used. The essential oils (EO) were prepared by conventional hydrodistillation (HD) and microwave-assisted hydrodistillation (MAHD). The volatile compounds of EOs were analyzed by gas chromatography spectroscopy (GC-MS). The antioxidant activities by means of DPPH radical scavenging and ferric-reducing antioxidant power (FRAP) were determined. The inhibition of tyrosinase activity was investigated. The cytotoxicity was performed against human fibroblast cell lines (NIH/3T3) and melanoma cell lines (A375 and B16F10). The decreasing melanin content was measured in melanoma cell lines. The resulting essential oils were detected for 41 compounds from HD extraction dominants by terpenes, namely sesquiterpenes (48.499%) and monoterpenes (19.419%), while 26 compounds were detected from MAHD with the fatty alcohols as the major group. The higher antioxidant activities were found in HD EO (IC50 of 16.25 ± 0.09 mg/mL from DPPH assay and 0.91 ± 0.01 mg TEAC/g extract from FRAP assay). The survival of normal fibroblast cell lines remained at 90% at 500 µg/mL HD EO, where the EO possessed the half-maximal toxicity dose (TD50) of 214.85 ± 4.647 and 241.128 ± 2.134 μg/mL on B16F10 and A375 cell lines, respectively. This could suggest that the EO is highly selective against the melanoma cell lines. The melanin content was decreased at the half-maximum efficacy (IC50) at 252.12 ± 3.02 and 253.56 ± 3.65 in the A375 and B1610 cell lines, respectively, which were approximately 2.8-fold lower than kojic acid, the standard compound. The results of this study evidence the use of *Etlingera elatior* (Jack) leaf as a source of essential oil as an active agent in cosmetics.

## 1. Introduction

Essential oils, also called volatile odoriferous oils, are obtained from many different plants, especially aromatic plants. The extraction of various plant parts, including flowers, buds, seeds, leaves, peels, and barks, vary in odor and flavor, resulting from the types and amounts of constituents present in oils [1]. The dominant constituents in essential oils can be divided into two major groups, terpene hydrocarbons and oxygenated compounds, which are terpenes (monoterpenes and sesquiterpenes), aromatic compounds (i.e., aldehyde, alcohol, phenol, methoxy derivative, and so on), and terpenoids (isoprenoids) [2]. Essential oils have been widely used as food flavors and are known to possess antioxidants [1,2]. The extraction method is one of the prime factors that determines the quality of essential oils. This can result in discoloration, off-odor/flavor, and physical changes, such as increased viscosity. The loss in bioactivity can be varied by the extraction conditions as well.

Essential oils can be extracted from plant materials by several methods; hydrodistillation, steam distillation, and expression are the conventional industrial-scale methods [3,4]. Hydrodistillation (HD) has become the standard method of essential oil extraction from plant material by the complete immersion of plant materials in water, followed by boiling. The surrounding water prevents the plant sample from overheating. The steam and essential oil vapor are condensed to an aqueous fraction. Modern technologies have been continuously developed to enhance extraction efficacy. Advanced technologies coupled with HD have been studied. Golmakani and Rezaei [5] studied microwave-assisted HD (MAHD), which is an advanced HD technique utilizing a microwave oven in the extraction process. MAHD was superior in terms of saving energy and extraction time [6].

*Etlingera elatior* (Jack) or Torch ginger belongs to the Zingiberaceae species. The inflorescence of it mean it has been used as an ornamental plant in Australia, Brazil, Hong Kong, Thailand, and the United States of America [6]. Jafar et al. compared the volatile compounds in leaf, stem, inflorescence, and rhizomes. The comparable yield of monoterpenes hydrocarbons, and a superior yield of sesquiterpene hydrocarbons, were found in the leaf over the inflorescence [7]. Wong et al. reported that the dominants of *E. elatior* leaf essential oil were mono- and sesquiterpenoids, with the major components being myrcene (13.5%), a-humulene (11.8%), b-caryophyllene (10.7%), camphene (18.0%), and b-pinene (16.9%) in the oils from the rhizomes and roots, respectively [8]. *E. elatior* species have been reported to possess significant biological activities such as antimicrobial, antioxidant, and antitumor activities [9]. Chan et al. [10] also reported the outstanding antioxidant properties of *E. elatior* leaves. Moreover, *Etlingera* species had the highest phenolic content and radical activity compared with 26 ginger species [11]. Whangsomnuek et al. reported the antioxidant activity of aqueous *E. elatior* flower and leaf extracts, as well as the inhibitory of tyrosinase and collagenase activities. It was concluded that the extracts were suitable for use as active ingredients for antiaging, antiwrinkle, and whitening purposes in cosmetic applications [12].

Therefore, in this study, we aimed to investigate the use of leaf as a source of essential oil. The extraction efficacy was compared between conventional HD and MAHD. The EE leaf essential oils were investigated for their yield, volatile compounds profiles, antioxidant activities, anti-tyrosinase activities, cytotoxicity, and the anti-melanogenesis property.

## 2. Materials and Methods

### 2.1. Plant Material

Torch ginger leaves at the mature stage with sizes of approximately 40–60 cm were purchased from Doi Ob Park (Chiang Rai, Thailand) in November–December 2021. Trolox ((±)-6-hydroxy-2,5,7,8-tetramethylchromane-2-carboxylic acid), kojic acid, epigallocatechin gallate (EGCG), DPPH (2,2-diphenyl-1-picrylhydrazyl), TPTZ (2,4,6-Tris(2-pyridyl)-s-triazine), L-DOPA (3,4dihydroxy-L-phenylalanine), and mushroom tyrosinase (EC 1.14.18.1) were purchased from Sigma-Aldrich (St. Louis, MO, USA). Minimum Essential Medium (MEM), Dulbecco’s Modified Eagle’s Medium (DMEM), fetal bovine serum (FBS), l-glutamine, penicillin, and streptomycin were purchased from ThermoFisher Scientific (Waltham, MA, USA).

### 2.2. Essential Oil Extractions

#### 2.2.1. Plant Preparation

Torch ginger leaves were washed with tap water, removed from the stem, shelter-dried, and freshly chopped. Before use, the sample was mixed with deionized water at 1:10 (*w*/*v*) ratio and blended for 5 min (HR2056, Philips, Bangkok, Thailand).

#### 2.2.2. Conventional Hydrodistillation

The extraction was performed in the 5 L round-bottom flask containing magnetic stirring bars for anti-bumping and heated at 150 °C (MS-E series, MTOP, Korea) for 4 h. The essential oil layer was collected, and the contaminant water was removed by the anhydrous salt and stored in the refrigerator until use.

#### 2.2.3. Microwave-Assisted Hydrodistillation

The microwave-assisted system (ETHOS^TM^ X, Milestone Srl, Milan, Italy) was used as the alternative hydrodistillation. The sample was subjected to the 5 L flask. The microwave power was set at 1700 watts for 4 h. The obtained essential oil was collected, and the contaminant water was removed by the anhydrous salt and stored in the refrigerator until use.

The obtained *Etlingera elatior* leaf essential oil was calculated for their percentage of yield using the below equation:

(1) %yield=(Amount of essential oil (g)×100)÷Amount of plant sample (g)

### 2.3. Volatile Compound Analysis of Essential Oil by GC-MS

The volatile compounds in essential oils were detected by Agilent 19091S-433 GC-MS equipment, equipped with HP-5MS column (30 m × 250 μm × 0.25 μm) (Agilent 19091S-433, Agilent Technologies, Santa Clara, CA, USA). Helium was used as a gas carrier (mobile phase) at a constant flow rate of 1 mL/min. In this program, the initial temperature was set at 60 °C, the final temperature was set at 240 °C, with an increasing rate of 3 °C/min, and held for 5 min [7]. The EO was prepared according to Wong et al. [8] with some modification by dissolving 10 mg of EO with 0.75 mL dichloromethane, then 1 μL of the sample was injected with a split ratio of 100:1. Relative quantities of the chemical compounds were expressed as percentages based on the peak area produced in the chromatogram. The relative content of each component was calculated according to peak area and compound, which specifies the identical comparison to mass spectra library search. Compounds annotated with higher than 90% similarity were reported.

### 2.4. Antioxidant Activities

#### 2.4.1. DPPH Radical Scavenging

The antioxidant efficiency of essential oils was investigated by DPPH assay [13]. Briefly, 0.1 mM DPPH solution was prepared in absolute ethanol. Various concentrations of EO from HD and MAHD were introduced to the DPPH solution. The reaction was mixed well and incubated in a dark ambient for 30 min. The DPPH radical scavenging ability was measured at a wavelength of 517 nm using a microplate reader (SPECTROstar Nano, BMG labtech, Aylesbury, UK) in triplicate. Trolox was used as a positive control. The inhibitory percentage of the DPPH radical scavenging ability was calculated against the control of DPPH solution without a sample. The IC50 value of each sample was obtained from the analytical curve.

#### 2.4.2. Ferric-Reducing Antioxidant Power

The ferric-reducing antioxidant power of EO was performed as per Pintathong et al. [14]. The FRAP reagent was freshly prepared prior the test by mixing 300 mM acetate buffer, 10 mM 2,4,6-Tris(2-pyridyl)-s-triazine (TPTZ in 40 mM HCl), and 20 mM ferric chloride solution in the ratio of 10:1:1 (*v*/*v*). Then, 20 µL of EOs were mixed with FRAP reagent (180 µL) and incubated at ambient for 5 min. After, the mixture’s absorbance was measured at 593 nm using a microplate reader (SPECTROstar Nano, BMG labtech, Aylesbury, UK). The experiment was performed in triplicate. Trolox was used as a positive control. The result was expressed as milligrams of Trolox-equivalent antioxidant capacity (TEAC) per gram of extract (mg TEAC/g extract).

### 2.5. Tyrosinase Inhibitory Activity

Mushroom tyrosinase inhibition was determined according to the previously described method with slight modifications [15]. Assays were conducted in a 96-well plate, L-DOPA was used as substrate, and kojic acid was used as a standard. Briefly, samples were dissolved in 25% DMSO in a phosphate buffer. In 96-well plates, 140 µL of the mixing of phosphate buffer (50 mM, pH 6.8) with various concentrations of EE essential oils was added. Kojic acid was used as a standard compound. Then, 10 µL of tyrosinase (50 U/mL) was added. After 10 min incubation, 50 µL of 0.95 mM L- DOPA was added and incubated for 10 min. The samples were analyzed by a microplate reader using absorbance at 475 nm using a SPECTROstar Nano microplate reader (BMG LABTECH). Each sample was accompanied by a blank that had all the components except L-DOPA. Results were compared with a control consisting of 25% DMSO in place of sample. The percentage of tyrosinase inhibition (TIP%) was calculated as follows:

(2) %Tyrosinase inhibition=(100−(Abs of control−Abs of sample treated)×100)÷Abs of control

### 2.6. Cytotoxicity

#### 2.6.1. Cell Culture

The NIH/3T3 (ATCC CRL-1658) fibroblast cell lines, human malignant melanoma cells A375 (ATCC^®^ CRL-1619™), and murine B16-F10 melanoma cells (ATCC^®^ CRL-6475™) were purchased from the American Type Culture Collection (ATCC, Manassas, VA, USA). Cells were cultured in Dulbecco’s modified Eagle’s medium (DMEM) containing 10% fetal bovine serum (FBS) and 1% (*v*/*v*) penicillin–streptomycin; they were maintained in a humidified atmosphere with 5% CO_2_ at 37 °C.

#### 2.6.2. Cytotoxicity

The cytotoxicity of EO was examined as described by Freshney [16], with some modifications. The cultured cell lines were seeded at the acquired density to obtain the monolayer of 80% confluence cells, with 1 × 10^4^ cells/mL for NIH/3T3, 3 × 10^4^ cells/well for B16F10, and 5 × 10^4^ cells/well for A375, in a 96-well plate. After 24 h of incubation in a CO_2_ incubator, cells were treated with various concentrations of EO. Trolox was used as a standard in NIH/3T3 cell lines and kojic acid in melanoma cell lines. Then, this was incubated for a further 24 h and MTT solution (0.25 mg/mL) was then added and incubated for another 4 h. The medium was discarded, and the purple MTT–formazan crystals were dissolved with 100 μL of DMSO and 25 μL of Sorensen’s glycine buffer. After 5 min, the reduction in soluble MTT to form water-insoluble formazan, absorbance at 570 nm was measured using a microplate reader (SPECTROstar Nano, BMG labtech, Aylesbury, UK). The experiments were performed in triplicate. The percentage of cell viability was calculated using the absorbance of treated cells against the untreated cells.

The selectivity index (SI) was calculated from the IC50 of the essential oil in normal fibroblast cells vs. melanoma cells to indicate the safety of EE essential oil application [17].

### 2.7. Measurement of Melanin Content

The melanin content was measured according to the previously described method with slight modification [18]. Briefly, 100 µL of cells were seeded in a 96-well plate at a density of 3 × 10^4^ cells/well for B16F10 and 5 × 10^4^ cells/well for A375. Then, they were incubated for 48 h for cell adhesion. The samples were then added and incubated for 24 and 48 h. After the solution in each well was removed, 100 µL of 1 M NaOH containing 10% DMSO was added and then incubated at 80 °C for 90 min. The absorbance was measured at 490 nm by using a microplate reader. Kojic acid solution was used as a standard for melanin content.

The obtained *Etlingera elatior* leaf essential oil was calculated for their percentage of yield, as follows:

(3) %Antimelanogenesis=(100−(Abs of control−Abs of sample treated)×100)÷Abs of control

### 2.8. Statistical Analysis

All experiments were performed in triplicate, and data were expressed as mean ± standard deviation (SD). We carried out the analyses via one-way analysis of variance (ANOVA), Tukey’s post hoc test (*p* = 0.05), and Student’s *t*-test, using the SPSS software program, version 22.0 (SPSS Inc., Chicago, IL, USA). The significant level was considered when *p*-values were less than 0.05 (*p* < 0.05).

## 3. Results

### 3.1. Extraction Yield of E. elatior Leaf Essential Oil

The obtaining EO from both HD and MAHD was light-yellow with a higher yield from MAHD than HD (0.099 ± 0.001% and 0.082 ± 0.001%, respectively). The higher extraction yield from MAHD in this study was a good agreement to the previous comparisons of HD and MAHD. From the *Thymus vulgaris* L. essential oil extraction by Golmakani and Rezaei, MAHD was evidenced to the rupture of essential oil glands with MAHD by scanning electron microscopy (SEM) [5]. The content of coriander seeds essential oil was (*v*/*w*), 0.325% and 0.31% for MAHD and HD, respectively [19]. The essential oils from mango flowers had higher yields from MAHD than HD (0.16 and 0.11%) [20], respectively.

Essential oils yield from MAHD was higher than HD, because the running process of distillation time from MAHD was shorter than HD, with no energy or temperature loss from ambient disturbance, and the sample reached boiling stage more rapidly. Compared with HD, with the longer process, the temperature from ambience was dissipated and diffused, leading to energy loss and a slow boiling stage [21].

### 3.2. Volatile Constituents of E. elatior Leaf Essential Oil

**Figure 1:**
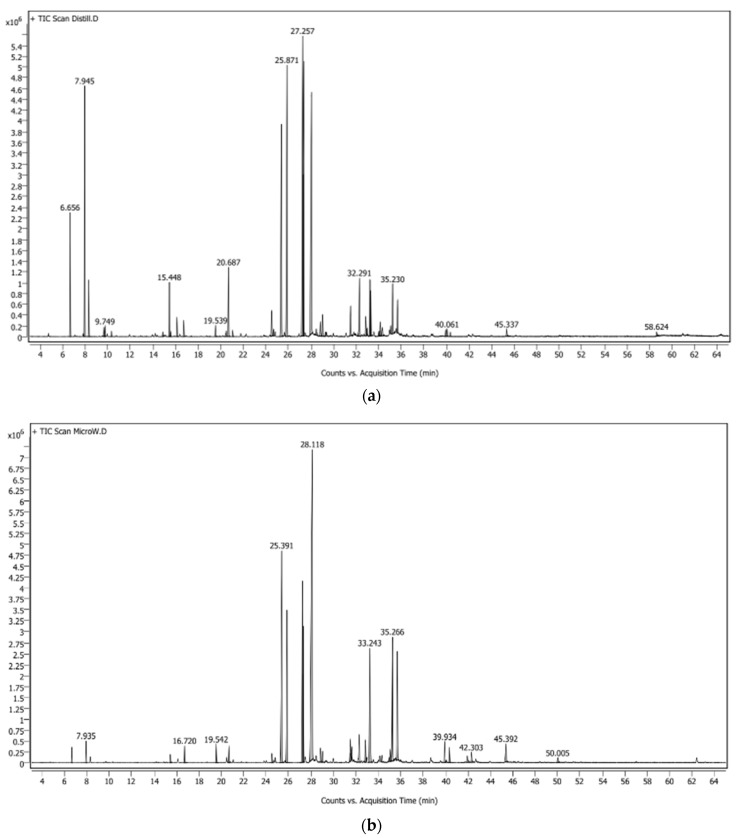
Gas chromatography chromatogram of *E. elatior* leaf essential oils from (**a**) hydrodistillation (HD) and (**b**) microwave-assisted hydrodistillation (MAHD).

A total of 41 volatile compounds were identified in the essential oils of the leaves of *E. elatior* by hydrodistillation (HD), and 26 volatile compounds were identified in the essential oils by microwave-assisted hydrodistillation (MAHD). The differences in number and percentage of each peak from total of volatile compounds in EO from HD and MAHD were found as the GC-MS profile in Figure 1 and Table 1. Figure 1 illustrates the significant differences in the present of small molecules in the early retention time in HD EO. The high force of the microwave destroys those small molecule volatile compounds. More obvious results can be seen in Table 1, where α- and β-pinene were significantly reduced when extracted with MAHD. The high force of the microwave can provide 9-Octadecyne, while this constituent is not found in the HD method. Chemical constituents such as β -pinene (7.354%), dodecanal (10.02%), cis-β-farnesene (10.55%), caryophyllene (12.56%), 1-dodecanol (13.48%), and humulene (15.21%) were the major compounds of the essential oil extract from HD, while dodecanal (12.42%) and 1-dodecanol (30.34%) were the major compounds in the essential oils extracted from MAHD.

The chemical constituents from torch ginger inflorescence essential oil were reported with the hydrocarbon as the major group constituents comprised ester (59.04%) and alcohol (39.95%), while for another group, terpenes (1.01%) [22]. The GC-MS showed that 1-dodecanol was found as a major component (23.89%) followed by lauryl acetate (21.51%). Mazlan et al. [23] also reported the strong antibacterial activity might be due to the high content of fatty alcohol (1-dodecanol) or presence of dodecanal (lauric acid). When the free carbonyl group of the fatty acid is reduced to corresponding aldehyde or alcohol, these compounds could be more effective than its acid [24]. The alcohols, ethers, ketones, aldehydes, and monoterpenes were reported for the antioxidant properties of essential oil [25]. Monoterpenoids and sesquiterpenoids are dominant compounds in EO of the leaves, roots, and rhizomes of *E. elatior*, the major compound such a-pinene (24.4%), humulene (7.2%) and b-pinene (6.6%) were dominant in the leaf oil. Moreover, non-terpenic aliphatic alcohols and aldehydes together accounted for 20.7% of the essential oils from the leaf, including dodecanol (9.9%) and dodecanal (6.2%) [8].

The volatile compounds in essential oils are highly affected by the extraction method. In this study, HD possessed higher monoterpenes and sesquiterpenes extraction efficacy than MAHD (19.42% and 2.66% vs. 48.50% and 27.75%), respectively. The previous study of mango flower essential oils from Wang et al. showed the higher monoterpene hydrocarbons, sesquiterpene hydrocarbons were obtained from HD. The oxygenated monoterpenes and other hydrocarbons such as alkanes and esters were obtained from MAHD [20]. Rosemary essential oils from MAHD and HD by Moradi et al. [25] were similar in profile, the higher monoterpene hydrocarbons were higher in HD, but oxygenated compounds and sesquiterpenes were higher in MAHD [25]. Okoh et al. [26] compared the HD with solvent-free MAHD. The monoterpene hydrocarbons were found in HD essential oil more than in the solvent-free MAHD extraction, while higher amounts of oxygenated monoterpenes were present in the oil extracted by solvent-free MAHD in comparison with HD [26]. The extraction of essential oils from *Anethum graveolens* L. (dill seed) and *Coriandrum sativum* L. (coriander seed) were altered by the use of MADH. Monoterpene hydrocarbon content of the MWHD oils was less than HD oils. Fatty acid content appeared to increase in coriander oils obtained by microwave energy [27].

### 3.3. Antioxidant Activities of E. elatior Leaf Essential Oils

The essential oils from HD possessed the higher antioxidant activities both in DPPH radical scavenging and ferric-reducing antioxidant power (FRAP) (Table 2). The results can be explained by the extracted volatile compounds mentioned before. The HD EO owns a higher number and higher amount of antioxidant volatile compounds; therefore, it exhibits a higher antioxidant activity. Tabana et al. [28] studied Sweet bay (*Laurus nobilis* L.) essential oil and its chemical composition, antioxidant activity, and leaf micromorphology under different extraction methods; the EO of Sweet bay (*Laurus nobilis* L.) from HD showed the highest antioxidant activity when compared with other extraction methods, namely HSD, MAHD, and OAHD. In a study reported by Bartikova et al. [29], higher DPPH scavenging from HD was related to the amounts of monoterpenes and sesquiterpenes present in the EO. However, the amount of antioxidant activity in oil from HD was higher than that from the MAHD method because the % composition of 1,8-cineol (eucalyptol) present in MAHD was higher than that of HD [30].

The differences in the antioxidative activity of different essential oils were mostly due to the differences in types and amounts of antioxidative components present in the essential oils [1,31]. Essential oils have several modes of antioxidant actions, such as through prevention of chain initiation, possession of free radical scavengers and reducing agents, termination of peroxides, prevention of continued hydrogen abstraction, as quenchers of singlet oxygen formation, and through binding of transition metal ion catalysts [32,33].

Antioxidant activity also varies with the source of essential oils. Tongnuanchan et al. [33] reported the antioxidant activities of Zingiberaceae root of *Zingiber montanum* and *Zingiber officinale* essential oils from Thailand and their application as antioxidants in food industries. The film with essential oils could increase the antioxidant activities by 50%. Thereafter, the essential oils from the leaves of various plants, namely lemongrass, basil, citronella, and kaffir lime, were also studied by Tongnuanchan et al. [34] for their antioxidant activity and their potential to be incorporated into antioxidant films for food antioxidant purposes.

### 3.4. Tyrosinase Inhibitory Activity

Tyrosinase inhibition activity can be analyzed using the modified dopa-chrome method with L-DOPA as the substrate. The IC50 of HD essential oil was 2.34 ± 0.04 mg/mL, while for MAHD it was 2.97 ± 0.03. Components from the GC/MS profile, such as terpenes, including α-pinene, β-pinene, α-terpineol, β-humulene, β-Farnesene, and β-myrcene, have been reported to exhibit anti-tyrosinase activities [35,36,37,38,39]. According to the research of Chan et al. [11], tyrosinase inhibition activity of methanolic *E. elatior* leaf extract is 55.2% at 0.5 mg/mL, which is higher than *E. fulgens* and *E. maingayi*, Zingiberaceae family plants. However, several anti-melanogenic agents are not direct tyrosinase inhibitors; instead, they modulate cellular signaling pathways to reduce the production of tyrosinase and its associated proteins, such as TRP-1 [40]. Therefore, the decreasing melanin content of HD oil was selected for the further determination.

### 3.5. Cytotoxicity

**Figure 2:**
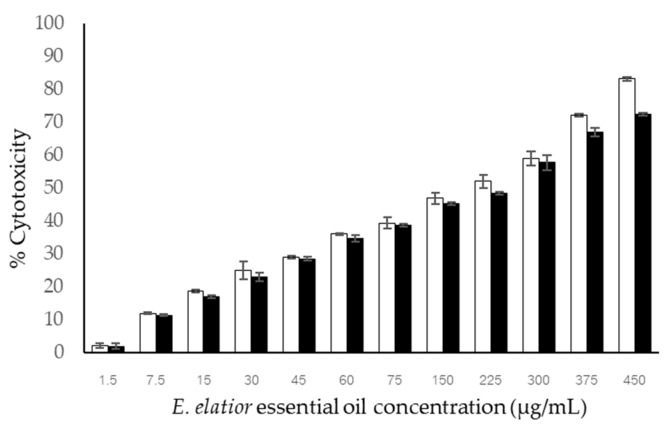
Melanoma cytotoxicity of *E. elatior* essential oil against B16F10 (□) and A375 (■) cell lines. Results are represented in mean percentage of cytotoxicity ± SD (*n* = 3).

The essential oil from the HD extraction method was selected to test for cytotoxicity against the fibroblast (NIH/3T3) and melanoma cell lines (A375 and B16F10). The viability was tested in the concentration range of 10–250 μg/mL. The result on NIH/3T3 cell lines demonstrated the safety range up to 500 μg/mL, at which more than 90% of cells survived [41]. According to the research of Prashar et al. [42], by the MTT test, less than 50–25% of cell survival was classified as moderately cytotoxic, and less than 25% was classified as highly cytotoxic. The maximum non-cytotoxic concentration (MNTC) was calculated as the concentration required to retained cell viability by 90% [42]. The previous studies on fibroblast cell cytotoxicity reported that the essential oil from *Origanum vulgare* possessed non-cytotoxicity against fibroblast cells (L929 cells) at concentrations up to 50 μg/mL, while 25 μg/mL of the sample showed 90% fibroblast cell viability [43]. The percentage of cell toxicity of EE EO against A375 melanoma and B16-F10 cells was concentration-dependent (Figure 2). The obtained IC50 of EE EO on B16F10 and A375 cell lines were 214.85 ± 4.647 and 241.128 ± 2.134 μg/mL, respectively. The standard compound of kojic acid showed no cytotoxicity against either melanoma cell lines (maximum concentration of 450 μg/mL). The recent study showed higher melanoma toxicity than the previous findings. *Tetradenia riparia* (Hochst.) Codd, *Bidens sulphurea* (Cav.) Sch. Bip., and *Foeniculum vulgare* Mill. essential oils possessed IC50 values on the B16F10 cell line of 272.37 ± 18.45, 230.00 ± 19.50, and 112.78 ± 13.74 μg/mL, respectively [44].

In summary, on cytotoxicity, the higher safety concentration for fibroblast cells (50 μg/mL) compared with the toxic concentration against melanoma cell lines (30 μg/mL) indicates the selective cytotoxicity of melanoma cells. The selective index cannot be calculated due to the undetectable IC50 value on fibroblast cell lines. The safety in use can be implied by the mentioned criteria.

### 3.6. Melanin Content

**Figure 3:**
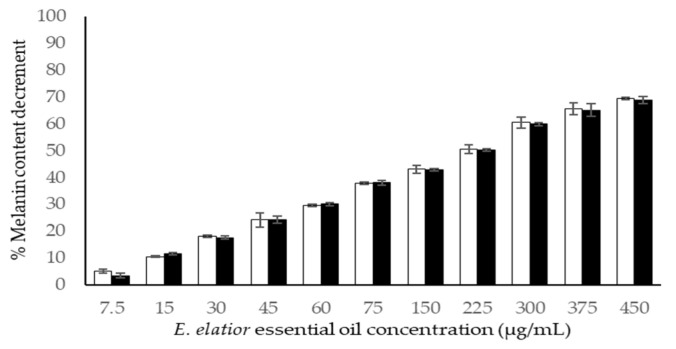
The reduction in melanin content by *E. elatior* essential oil against B16F10 (□) and A375 (■) melanoma cell lines. Results are represented in mean percentage of cytotoxicity ± SD (*n* = 3).

The inhibition of melanin content by EE EO and kojic acid are illustrated in Figure 3. The inhibition of melanin content by EO was concentration-dependent against both cell lines. Besides human melanoma cell lines, mouse melanoma cell lines of B16F10 have been extensively used in the investigation of the melanogenesis effects of natural extracts [45,46]. The EO exhibited a 50% melanin content decrement at 252.12 ± 3.02 and 253.56 ± 3.65 in the A375 and B1610 cell lines, respectively. The positive standard compound of kojic acid possessed 89.33 ± 4.04 and 87.79 ± 4.00 μg/mL in the tested cell lines, respectively. It can be summarized that the EE EO showed three times less efficacy than the standard anti-melanin compounds.

From the recent results of chemical compositions, the compounds which demonstrate the anti-melanogenesis candidates in EE EO are α-terpineol, β-myrcene, α-pinene, β-pinene, β-caryophyllene or β-humulene, and bornyl acetate, because they have been reported for the reduction in melanin content in [35,36,38,47].

## 4. Conclusions

This study revealed the comparable *E. elatior* leaf essential oil extraction efficacy of conventional HD. The GC/MS profiles illustrated the dominants of terpenes, namely sesquiterpenes (48.499%) and monoterpenes (19.419%). The antioxidants, by means of DPPH radical scavenging and ferric-reducing antioxidant power, were superior in essential oils from HD compared with MAHD. The cytotoxicity was found more selective in melanoma cell lines than in fibroblast cell lines, illustrating preliminary safety in topical uses. It was highlighted that the melanin content in melanoma cell lines was decreased after being treated with EO. It can be concluded that the *E. elatior* leaf can be value-added by being a source of essential oils for cosmetic fragrances and as an active ingredient. Further study on multiple skin-related cell lines, as well as clinical trials on skin efficacies, should be carried out to confirm the uses of essential oils in topical preparations.

## Figures and Tables

**Table 1:** Volatile constituents of *E. elatior* leaf essential oils from hydrodistillation (HD) and microwave-assisted hydrodistillation (MAHD).

	Compounds	CAS No.	HD	MAHD
**Aldehyde**	Decanal	112-31-2	0.556	0.705
	Undecanal	112-44-7	0.203	-
	Dodecanal	112-54-9	10.024	12.422
**Alkene**	9-Octadecyne	35365-59-4	-	0.725
**Carboxylic ester**	9-Tetradecen-1-ol, acetate, (E)-	23192-82-7	0.249	0.919
	1-Tetradecyl acetate	638-59-5	0.334	0.669
	Lauryl acetate	112-66-3	2.245	6.599
**Dialkyl ketone**	2-Undecanone	112-12-9	0.274	-
**Fatty acids**	Dodecanoic acid	143-07-7	0.542	-
	Hexadecanoic acid	57-10-3	0.334	1.136
**Fatty aldehyde**	cis,cis-7,10,-Hexadecadienal	56829-23-3	0.351	-
	7-Tetradecenal, (Z)-	65128-96-3	-	1.084
	Pentadecanal-	2765-11-9	0.264	-
**Fatty alcohols**	1-Decanol	112-30-1	0.425	0.802
	1-Dodecanol	112-53-8	13.484	30.335
	11-Hexadecen-1-ol, (Z)-	56683-54-6	-	1.094
	cis-9-Tetradecen-1-ol	35153-15-2	1.989	6.522
	1-Tetradecanol	112-72-1	1.224	5.711
**Monoterpenes**	α-Pinene	80-56-8	3.055	0.443
	(±)-β-Pinene	127-91-3	7.354	0.707
	β-Myrcene	123-35-3	1.524	-
	(+)-m-Mentha-1(6),8-diene	1461-27-4	0.291	-
	Eucalyptol	470-82-6	0.312	-
	trans-β-Ocimene	3779-61-1	0.154	-
	Pinocamphone	547-60-4	0.162	-
	3-Pinanone	15358-88-0	1.985	0.358
	Terpinen-4-ol	562-74-3	0.152	-
	α-Terpineol	98-55-5	0.692	-
	Methyl myrtenate	30649-97-9	2.674	0.711
	(+)-3-Carene, 10-(acetylmethyl)-	163886-28-0	1.064	0.438
**Sesquiterpenes**	β-Elemene, (−)-	515-13-9	0.245	-
	Caryophyllene	87-44-5	12.575	7.857
	Humulene	6753-98-6	15.207	9.852
	(E)-β-Farnesene	18794-84-8	-	5.747
	cis-β-farnesene	28973-97-9	10.546	-
	β-Chamigrene	18431-82-8	0.298	-
	α-Bergamotene	17699-05-7	0.685	-
	6-Methyl-5-hepten-2-ol	58319-05-4	-	0.726
	Isodaucene	142878-08-8	0.862	0.531
	Germacrene B	28387-44-2	0.157	-
	α-Nerolidol	40716-66-3	1.352	1.185
	Caryophyllene oxide	1139-30-6	2.823	1.515
	Ledol	577-27-5	0.881	-
	humulol	28446-26-6	0.225	-
	Humulene epoxide II	19888-34-7	1.559	-
	Humulenol-II	19888-00-7	0.611	
	Caryophylla-3(4),8-dien-5-ol	19431-79-9	0.473	0.34
**Total**	Aldehyde		10.783	13.127
	Alkene		0	0.725
	Carboxylic ester		2.828	8.187
	Dialkyl ketone		0.274	0
	Fatty Acids		0.876	1.136
	Fatty aldehyde		0.615	1.084
	Fatty alcohol		17.122	44.464
	Monoterpenes		19.419	2.657
	Sesquiterpenes		48.499	27.753

The results are presented as area sum, %.

**Table 2:** Antioxidant activities of *E. elatior* leaf essential oils from hydrodistillation (HD) and microwave-assisted hydrodistillation (MAHD).

	HD	MAHD
DPPH (IC50, mg/mL)	16.25 ± 0.09 ^a^	39.29 ± 0.27 ^b^
FRAP (mg TEAC/g)	0.91 ± 0.01 ^a^	0.43 ± 0.00 ^b^
Tyrosinase inhibition (IC50, mg/mL)	2.34 ± 0.04 ^a^	2.97 ± 0.03 ^b^

The superscribed small letter displayed the significant differences (*p* < 0.05) between the extraction methods.

## Data Availability

Not applicable.

## Sample Availability

Samples of the compounds are not available from the authors.
